# Non-equilibrium electron transport induced by terahertz radiation in the topological and trivial phases of Hg_1−_*_x_*Cd*_x_*Te

**DOI:** 10.3762/bjnano.9.96

**Published:** 2018-03-29

**Authors:** Alexandra V Galeeva, Alexey I Artamkin, Alexey S Kazakov, Sergey N Danilov, Sergey A Dvoretskiy, Nikolay N Mikhailov, Ludmila I Ryabova, Dmitry R Khokhlov

**Affiliations:** 1 Physics Department, M.V. Lomonosov Moscow State University, Leninskie Gory 1 bld.2, 119991 Moscow, Russia; 2 Faculty of Physics, University of Regensburg, Universitaetstr. 31, D-93053 Regensburg, Germany; 3 Rzhanov Institute of Semiconductor Physics, pr. Lavrentieva 13, 630090 Novosibirsk, Russia; 4 Chemistry Department, M.V. Lomonosov Moscow State University, Leninskie Gory 1 bld.3, 119991 Moscow, Russia; 5 P.N. Lebedev Physical Institute, Leninskiy prosp. 53, 119991 Moscow, Russia

**Keywords:** terahertz radiation, topological insulator, photoconductivity

## Abstract

Terahertz photoconductivity in heterostructures based on n-type Hg_1−_*_x_*Cd*_x_*Te epitaxial films both in the topological phase (*x* < 0.16, inverted band structure, zero band gap) and the trivial state (*x* > 0.16, normal band structure) has been studied. We show that both the positive photoresponse in films with *x* < 0.16 and the negative photoconductivity in samples with *x* > 0.16 have no low-energy threshold. The observed non-threshold positive photoconductivity is discussed in terms of a qualitative model that takes into account a 3D potential well and 2D topological Dirac states coexisting in a smooth topological heterojunction.

## Findings

Discovery of theoretically predicted quantum spin Hall effect states in HgTe quantum wells [1–2] has initiated extensive studies of topological insulator materials [3–4]. Noteworthy, the ARPES technique, being a well-developed method to probe topological surface states, is a challenge in the case of HgTe-based topological insulators due to its zero-gap energy spectrum in the bulk. Nevertheless, formation of topological surface states in 3D HgTe has been convincingly proved by ARPES experiments in several detailed studies [5–7].

Hg_1−_*_x_*Cd*_x_*Te solid solutions demonstrate a composition-driven transition from the topological phase with inverted band structure to the trivial phase with normal band structure ordering at *x* ≈ 0.16 [8]. In contrast to most of the 3D topological insulators, Hg_1−_*_x_*Cd*_x_*Te solid solutions are characterized by relatively low free carrier concentration values in the bulk, and may be therefore considered as good candidates for a case study focused on determination of the topological state contribution to the charge carrier transport. Laser terahertz probing is known to be a powerful tool that may provide an insight into the electron dynamics in semiconductors, particularly, in topological insulators [9–11]. Study of non-equilibrium processes in Hg_1−_*_x_*Cd*_x_*Te in the terahertz spectral range is additionally motivated by the application aspects related to the terahertz photodetector development [12].

In our recent paper [13], we have shown that photoconductivity in Hg_1−_*_x_*Cd*_x_*Te solid solutions at 280 µm wavelength changes its sign across the topological transition from the inverted to the normal band structure. It was assumed that the negative photoresponse in the samples with the normal band structure is most likely related to the electron gas heating, while the positive photoconductivity in the zero band gap mercury cadmium telluride was reasonable to associate with interband transitions.

In this work, we focus on the study of terahertz photoconductivity in the spectral range of 90–496 µm of Hg_1−_*_x_*Cd*_x_*Te solid solutions in close vicinity of the band inversion point. This is done to determine possible effects of the topological states on the non-equilibrium transport.

**Figure 1:**
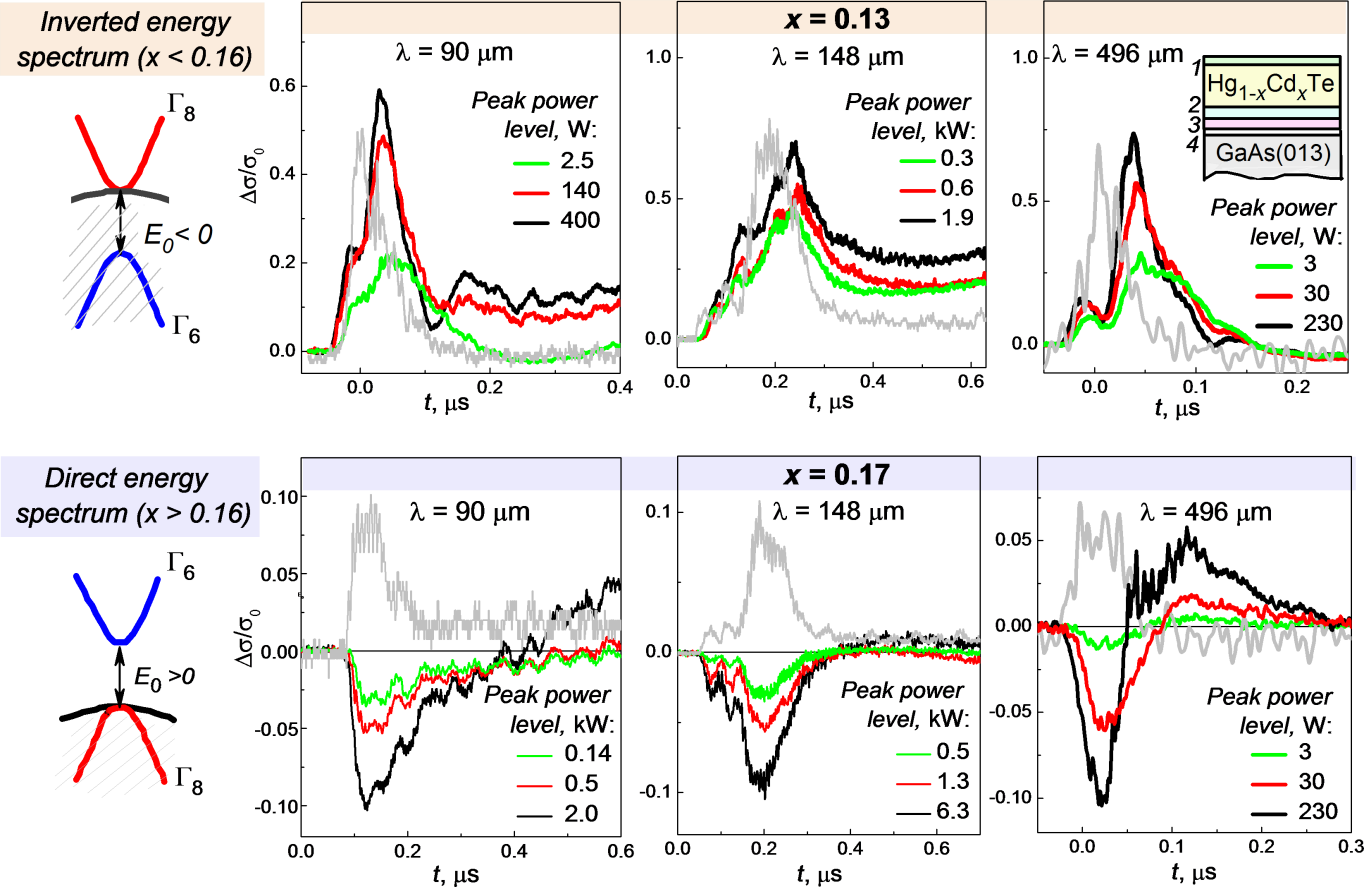
Photoconductivity kinetics Δσ/σ_0_ in Hg_1−_*_x_*Cd*_x_*Te films with *x* = 0.13 (the upper panel) and *x* = 0.17 (the lower panel) at the wavelengths λ = 90; 148; 496 µm for various radiation peak power levels. The laser pulse time profiles are shown by grey lines. The energy band structure for both solid solutions is shown schematically to the left of the plots. The heterostructure layers are outlined in the right upper corner. The cap and relaxed Hg_1−_*_y_*Cd*_y_*Te, buffer CdTe and ZnTe layers are indicated by the numbers from 1 to 4, respectively.

The Hg_1−_*_x_*Cd*_x_*Te heterostructures were synthesized by MBE. ZnTe and CdTe buffer layers, a CdTe-rich mercury cadmium telluride relaxed layer, a 3D Hg_1−_*_x_*Cd*_x_*Te layer, and a CdTe-rich cap layer were successively grown on a GaAs (013) semi-insulating substrate (see the inset in upper right corner of the Figure 1). The active 3D Hg_1−_*_x_*Cd*_x_*Te layer thickness was about 4 µm. Composition of the films was controlled by ellipsometry. The synthesis is described in detail in [14].

We have chosen samples with *x* = 0.13; 0.15; 0.17 for our study. The latter corresponds to the trivial phase with the normal band structure. The two others are characterized by the inverted band structure (topological phase). Hall effect measurements have shown that all the samples are of the n-type. Free electron concentration values determined in magnetic field of 0.05 T at *T* = 4.2 K are in the range from 3.7 × 10^14^ cm^−3^ to 5.2 × 10^14^ cm^−3^. Within the two-band Kane model, the given concentrations correspond to the Fermi level position not lower than at 3 meV, 5 meV, and 7 meV above the conduction band edge for the samples with *x* = 0.13, 0.15, 0.17, respectively. The energy distance between the conduction band and the light-hole valence subband used in the Kane model calculations was estimated using the empirical relations [15–20].

Photoconductivity kinetics has been studied under 90, 148, 280, and 496 µm wavelength pulse laser radiation at the temperature 4.2 K. The measurements have been done in the Hall bar geometry using the 4-probe method. The incident radiation was normal to the sample surface. Duration of the pulse was ≈100 ns. The radiation power was up to 7 kW and could be varied by calibrated attenuators. The use of the incident radiation power as a variable parameter can help to figure out mechanisms of the photoelectric phenomena in some cases [21–22]. The experimental details can be found elsewhere [23–27].

The photoconductivity kinetics Δσ/σ_0_ for the samples with *x* = 0.13 (the upper panel) and *x* = 0.17 (the lower panel) is shown in the Figure 1. Here Δσ is the change in conductivity under pulse irradiation, σ_0_ is the conductivity value before the laser pulse. The data for the structures with *x* = 0.13 and *x* = 0.15 (inverted band structure) are quite similar, therefore only data for the sample with *x* = 0.13 are presented in the Figure 1. The observed kinetics are rather complicated and can be described by several superimposed processes characterized by different relaxation time parameters. We will address here only to relatively fast processes with the characteristic times of 100–200 ns. The long-term photoconductivity observed at longer times after the laser pulse end may be due to photoinduced transitions to or from the local electron states in the barriers. This long-term photoconductivity is not discussed in this paper. It is important that the signs of the fast photoresponse for the normal and inverted band structure samples are opposite. For the latter, the photoconductivity is positive. Beside that, it demonstrates certain time delay with respect to the excitation laser pulse. The negative photoconductivity in the normal band structure case is much smaller in amplitude, and its kinetics repeats the laser pulse time profile.

**Figure 2:**
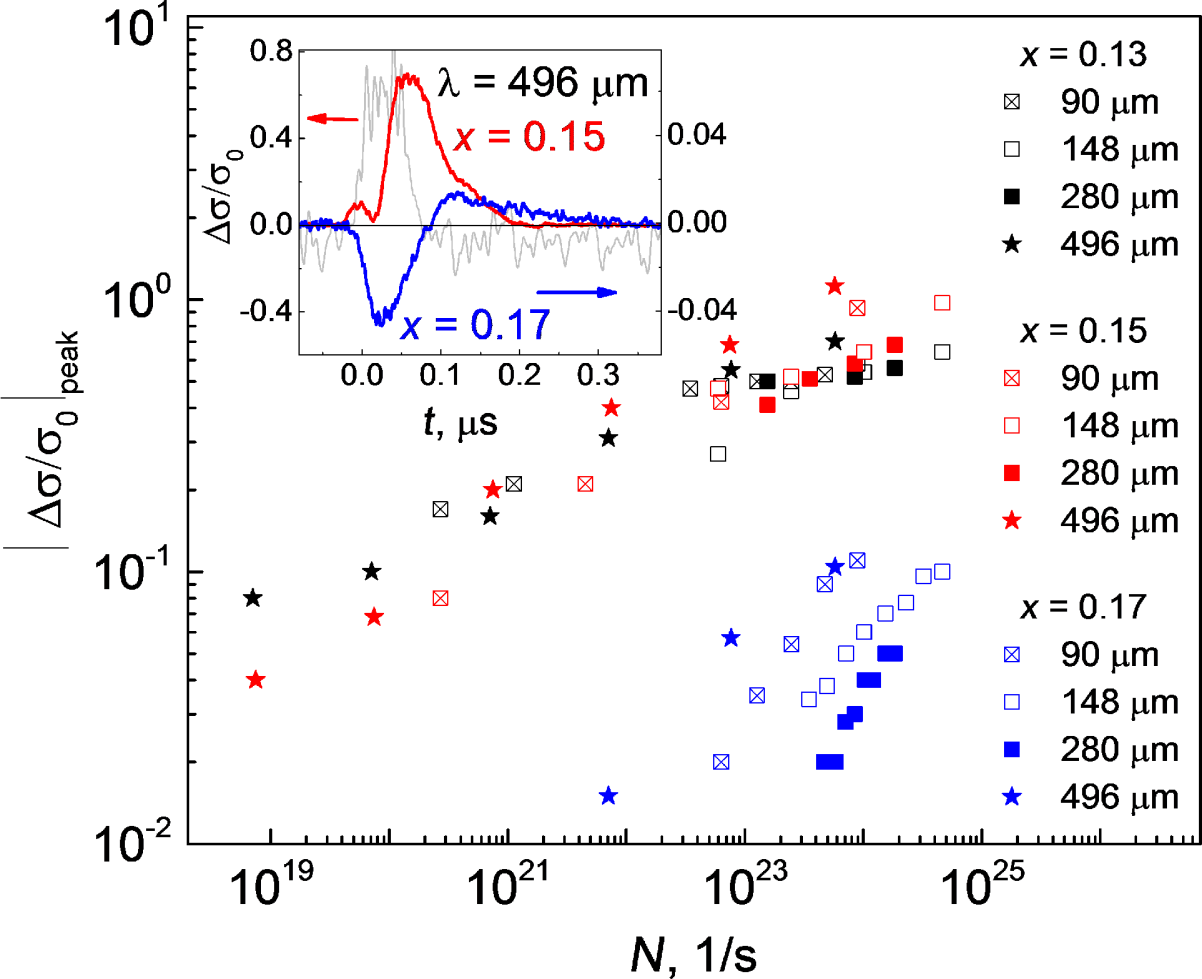
Dependence of the absolute value of the peak photoresponse amplitude |Δσ/σ_0_|_peak_ (σ_0_ is the conductivity before a laser pulse, Δσ is the conductivity change under illumination) on the photon flux density *N* for Hg_1−_*_x_*Cd*_x_*Te films with *x* = 0.13 (black symbols), *x* = 0.15 (red symbols), and *x* = 0.17 (blue symbols) at various wavelengths. The photoconductivity kinetics at 496 µm for the samples with *x* = 0.15 and *x* = 0.17 are shown in the inset. A typical pulse time profile is shown by the grey line.

Photoconductivity kinetics keeps the features mentioned above at lower radiation power levels (Figure 1). The absolute value of photoresponse amplitude │Δσ/σ_0_│_peak_ versus the number of the incident quanta *N* per unit time is shown in the Figure 2 for all wavelengths used and all samples studied. The photoconductivity amplitude dependence on the photon flux *N* for the samples with *x* = 0.13 and *x* = 0.15 is nonlinear and may be well fitted by the power dependence │Δσ/σ_0_│ ~ *N*^α^, where α is close to 1/4. It is important that the experimental data corresponding to the samples with *x* = 0.13 and *x* = 0.15 are close for all wavelengths used. It means that the photoconductivity value is defined only by the incident photon flux *N* irrespectively of the wavelength. It is reasonable to assume therefore that the positive photoconductivity in these samples results from an increase in the free carrier concentration due to the photogeneration process with the constant quantum efficiency independently on the wavelength. It should be stressed that the positive photoconductivity is still observed even for the sample with *x* = 0.15 for which the Fermi energy (>5 meV) well exceeds the quantum energy of the 496 µm wavelength laser radiation (2.5 meV).

In contrast to that, the negative photoconductivity (sample with *x* = 0.17) depends strongly on the radiation wavelength. The electron gas heating by the incident radiation followed by an electron mobility drop is most likely responsible for this effect. This mobility drop is due to a scattering time drop with increasing energy, as well as to a substantial increase in the electron effective mass of hot electrons. This process obviously has no energy threshold. In such a case, the photoconductivity is negative and depends on the power absorbed. Therefore, the data calculated as a function of the incident quantum flux (see Figure 2) differ for different wavelengths. An additional discrepancy may come out as a result of carrier trapping by acceptor resonant states [28–29].

Let us discuss now in more detail the experimental results obtained for the Hg_1−_*_x_*Cd*_x_*Te topological phase (*x* < 0.16). The most unusual result is the absence of a threshold energy in the strong generation-related positive photoconductivity. The photoresponse is observed even if the Fermi energy exceeds the energy of the incident radiation quantum. Existence of the topological heterojunction may be a key factor that determines the non-threshold photoexcitation in the structures studied.

**Figure 3:**
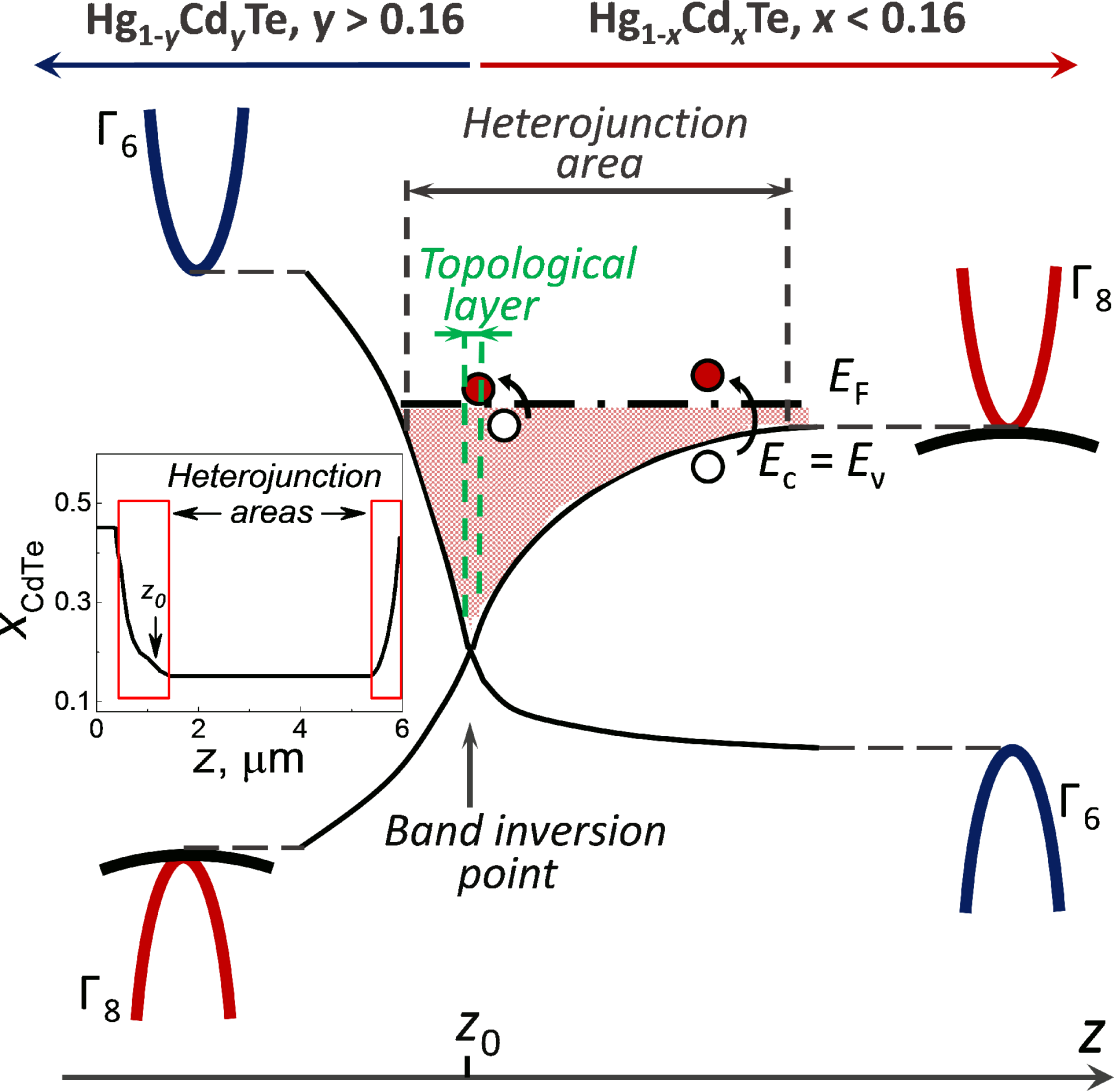
Sketch of the smooth heteroboundary between the Hg_1−_*_x_*Cd*_x_*Te active layer (with the inverted band structure) and the Hg_1−_*_y_*Cd*_y_*Te barrier layer (with the normal band structure) in the samples with *x* < 0.16. Variable position edges of the conduction (*E*_c_) band, the heavy hole valence (*E*_v_) subband, and the light hole subband in the heterojunction are schematically shown by black solid lines. The Fermi level is shown by the dash-dot line. The topological layer located in the close vicinity to the *z*_0_ position is sketched up by green dashed lines. The red and the blank circles shown above and below the Fermi level, respectively, correspond to the suggested mechanisms of the positive photoconductivity effect. The CdTe content along the heterostrocture profile is presented in the inset. The red rectangles correspond to the heterojunction areas, the left one of which is zoomed in the main part of the figure.

Indeed, the buffer and cap layers of the heterostructure are formed of Hg_1−_*_x_*Cd*_x_*Te solid solutions with a relatively high CdTe content providing normal band structure ordering. The film under study is in the topological phase with the inverted relative positions of the conduction and light hole bands. The CdTe content *x* varies quite smoothly on the characteristic length of about 1 µm along the heterojunctions between the buffer and the film, as well as between the film and the cap layer. Previously, it was theoretically demonstrated that in such a situation, there should appear a 3D potential well in the heterojunction area [30–32]. Beside that, 2D topological Dirac states are formed at the position *z*_0_ corresponding to the gap absence between the conduction and light hole bands (Figure 3). To the right of *z*_0_, the bulk semiconductor energy spectrum is gapless. The Fermi level position in such a structure varies with respect to the potential well bottom along the heterostructure profile. Therefore, for any given energy of a terahertz quantum, there should exist a position in the heterojunction area for which photogeneration from the heavy hole band to the conduction band becomes possible. It is important that this generation process has no threshold in energy, and its intensity is defined by the number of incident radiation quanta. Therefore, it may give rise to the positive photoconductivity observed experimentally.

There is one more possible mechanism providing appearance of the positive photoconductivity in heterostructures under study. As it was mentioned earlier, 2D Dirac states are formed at the position *z*_0_ corresponding to the bottom of the 3D heterojunction potential well. Heating of electrons by the incident terahertz radiation in the 3D well leads to two competing effects. The first one is the mobility drop that should result in the negative photoconductivity. The second effect corresponds to the spatial diffusion of excited electrons to the 2D area. Indeed, it is located at the bottom of the well. Beside that, the density of the 2D Dirac states depends linearly on energy *E*, whereas it is proportional to *E*^1/2^ for the bulk conduction band states. It means that for the heated electrons, there is an increased probability to diffuse to the *z*_0_ position. Mobility of 2D Dirac electrons is much higher than it is for the bulk electrons, therefore this diffusion process results in the positive photoconductivity. The amplitude of this effect is much higher than the mobility drop due to the electron gas heating, so the positive photoconductivity prevails. Moreover, the diffusion process is delayed with respect to the photoexcitation which is observed experimentally. This is due to the fact that the spin direction of 2D Dirac electrons is locked to their momentum vector direction, whereas the 3D electrons in the well do not possess this feature. The suggested mechanism for the positive photoconductivity is non-threshold in energy. As a final argument, the 3D potential wells, as well as 2D Dirac states should not be formed for the Hg_1−_*_x_*Cd*_x_*Te films with the composition corresponding to the trivial phase, and the positive delayed photoconductivity is not observed for these structures.

The two mechanisms for the positive photoconductivity suggested above may coexist in the same structure.

In summary, we have observed a non-threshold positive photoconductivity in heterostructures based on Hg_1−_*_x_*Cd*_x_*Te thick films being in the topological phase. We suggest possible mechanisms responsible for the effect that takes into account diffusion of photoexcited electrons in the heterojunction area to the 2D Dirac state.
